# “I am a survivor!”: Violently Injured Black Men’s Perceptions of Labeling After a Violent Firearm Injury

**DOI:** 10.1007/s11524-024-00874-8

**Published:** 2024-05-20

**Authors:** Nazsa S. Baker, Cortney VanHook, Devon Ziminski, Jordan Costa, Michael Mitchell, Nakita Lovelady

**Affiliations:** 1 School of Public Health, New Jersey Gun Violence Research Center, Rutgers University, 683 Hoes Lane West, Piscataway, NJ 08854 USA; 2 School of Social Work, University of Illinois- Urbana-Champaign, Urbana, IL USA; 3 School of Social Work, Rutgers University, New Brunswick, NJ USA; 4 School of Criminal Justice, Rutgers University, Newark, NJ USA; 5 School of Humanities and Social Sciences, The College of New Jersey, Ewing Township, NJ USA; 6 College of Public Health, University of Arkansas Medical Sciences, Little Rock, AR USA

**Keywords:** Victim, Survivor, Qualitative, Black men, Firearm violence, Injury

## Abstract

Self-appraisal after a life-altering event is a critical process for individuals, often comprised by assigned labels that may not align with an individuals’ perceptions of themselves or of their situation. Existing research within this victim-survivor dichotomy largely rests in the interpersonal violence space, with a victim assuming legal recourse and wrongdoing, and a survivor associating with positive personal characteristics like grit and resilience. Much existing literature on self-appraisal after interpersonal injury is heavily concentrated within the sexual violence literature, and this study applies these concepts to a sample of Black men injured by firearms. Ten Black men enrolled in a hospital-based violence intervention program (HVIP) were interviewed to understand how they label their experience of firearm injury, and if their perceptions aligned with common labels seen among other populations and/or in other areas of study (e.g., cancer, domestic violence). Each participant assigned themselves their own label, with three labels emerging: survivor, victim and survivor, and neither victim nor survivor. The results illustrate the nuance of experiences beyond the victim-survivor dichotomy, and how labels and personal identities may shift following injury into new terms and considerations of resilience and trauma processing. More research is warranted to understand the factors that shape self-labeling within this population, including influences of masculine norms, racialized stereotypes, community context, and availability of services. Findings support public awareness campaigns to reframe surviving violence as a strength, and for community partners and practitioners to increase access to culturally competent and trauma-informed mental healthcare.

## Background

Language sensitivity is an important aspect of effective scholarship and service provision [1]. Labels can reinforce stigmatizing stereotypes of affected groups and can provoke psychological reactions among group members [2]. At times, affected groups use or prefer different labels than the ones ascribed to them by others, potentially leading to additional dissonance [2]. It is essential that health care and service providers, law enforcement, policymakers, and social scientists engage in language sensitivity for groups that have psychological and social vulnerabilities.

Subpopulations across the U.S. have experienced varying degrees of naming and labeling that affect self-concept, particularly Black men. Black men with a firearm-related injury have intersecting identities that require language sensitivity. Masculine socialization requires that men project an image of strength, control, and agency [3]. Historically, however, Black men in the United States have been figuratively and literally locked out of traditional heteropatriarchy (e.g., being a provider and protector of the family) through processes of enslavement and its continuing legacy today [4]. For example, this legacy can be seen in labor market participation where Black men face discrimination and are less likely than white men with prior felony convictions to receive callbacks from prospective employers [5]. Furthermore, for Black men living in resource-deprived communities, the hyper-policing, surveillance, and criminalization they are often subjected to creates a dilemma that renders them powerless in the eyes of the state [6, 7]. Consequently, the pathway to traditional masculinity for some Black men is “one of punitiveness” [8]. Black men are also exposed to racialized gendered socialization that minimizes their vulnerabilities and ascribes to them supernatural physical strength and mental resiliency [9]. Black men have systemically been exposed to a range of labels forced upon them through problematic and racialized structures that have presented them as torpid and unintelligent [10–14].

As the U.S. population most disproportionally affected by one of the most pressing public health crises of our time, firearm violence, Black men with a firearm-related injury have intersecting self-identities, compounded by centuries of external labels, that require language sensitivity. Multiple prior studies have qualitatively explored the experiences of Black men who have experienced a firearm injury, focusing on their experiences within the health care system, their access to care and recovery process following injury [15–17]. Hink et al. study with Black and white participants explored perceived injury risk, sense of safety, and firearm ownership before and after the injury [18]. Prior research has pointed to the onset or deepening of psychological stressors/triggers post-injury [15, 19] and daily experiences of and exposures to violence entrenched by designed structural inequities. The experience of firearm-injury is both deeply personal and driven by decades of intentional disinvestment and other structural and policy factors and can therefore be stigmatizing and harmful to the self-concept of Black men.; however, research has yet to more deeply explore language associated with Black men who experience a firearm injury, both external labels and self-identifiers.

*Victim* is a label conventionally applied to a person who experienced interpersonal violence [20]. Victim labelling acknowledges wrongful, illegal, and harmful acts committed by one person against another. Additionally, the label of victim generally produces action towards social and legal recourse [20]. Victims of domestic violence, sexual assault, and war crimes have successfully used the label of victim to achieve social change as well as civil and criminal punishment of perpetrators [21, 22]. On the other hand, *Survivor* has gained popularity over the past few decades as a form of psychological healing to aid individuals who have experienced life threatening or life altering events [23, 24]. Survivor has often been associated with personal characteristics such as resiliency, agency, active resistance, and recovery [20]. The *victim-survivor paradox* reflects the tension between the need to consider recourse stemming from the violent event and the need to cope and overcome the violent event [25].

Prior research indicates patterns of self-appraisal after interpersonal violence. Identification as a survivor is associated with positive mental health outcomes whereas victim identification has been associated with negative mental health outcomes such as shame, PTSD, and depression [26, 27]. What possibly distinguishes survivors from victims is survivors externalize their feelings which facilitates action whereas victims internalize feelings which contribute to immobility and silence [26]. Self-appraisal after interpersonal violence varies due to social attitudes that may be either supportive or harmful to one’s self-concept [23]. Survivors have typically received supportive responses whereas victims have been shamed [23]. Regardless of existing labels or general terms applied to them, both, victims and survivors can and do make sense of their own experiences that differ from socially constructed meanings. Several studies have found that victims and survivors had similar reactions (i.e., psychological reactions, agency, and appraisal) after injury [26–28]. Further, some choose to not utilize the dichotomous appraisal of their experiences and either reject labels themselves or apply an individualized label [26–29]. Additionally, self-appraisal is not a static process. A victim can transition to identifying as a survivor and a survivor, upon a commitment to reflection and healing, may come to acknowledge harm experienced and seek recourse. Language portraying victim/survivor labels as a spectrum have more recently arisen, relying on the lead of the person who experienced a potentially life altering event to self-appraise in ways that are most authentic to them [30].

Literature on self-appraisal after interpersonal injury is heavily concentrated within the sexual violence literature, and among people who identify as women [31, 32]. There is a need to understand self-appraisal among those injured by firearm violence. Firearm-related injury is a public health emergency, affecting over 85,000 people each year in the United States [33]. Some populations, such as Black men, experience firearm-related injury disproportionately [34]. The few existing studies on language and firearm injury address moving away from racially coded language (e.g., using “reinjury, recurrent injury, repeat victimization, and/or risk of repeated injury” as opposed to “recidivism” among those injured by firearms) [35]. Recent work by Betz et al. [1], discusses avoiding the use of the language “innocent victims” because the words imply that some people deserve to get shot. The alternative consideration is that all victims are equally deserving of medical care and prevention efforts. While other research on individuals who experienced nonfatal firearm injuries referred to participants as victims and/or victims and patients/participants [14, 17, 19]; as both survivors and victims’ [15, 18]; or as survivors [16] they did not identify the definition or meaning of victim or survivor within the study’s context, nor identify if the language used was ascribed to participants or self-identified by participants.

Black men experience firearm injury and death at an alarming and disproportionate rate. The continued injury warrants further research that forefronts the critical insight of the population(s) most affected, and in ways that both support Black men’s self-identification of their own experiences and highlight the structural inequities that contribute to the contexts and environments in which firearm injuries occur. Research illustrating the Black male voice on the survivor/victim spectrum within firearm injury fills a gap in research knowledge by contributing lacking evidence around this topic in the gun violence literature (as much to date exists in the cancer and intimate partner violence spaces); centers the individuals most intimately affected; and provides insight into language choices that can be used in clinical situations to ultimately support self-selected language across all domains – law enforcement, clinical settings, research/academic, and practitioner spaces. Therefore, for this study, ten Black men enrolled in a hospital-based violence intervention program (HVIP) were interviewed to understand how they perceive survivor and victim labeling after experiencing a firearm-related injury.

## Methods

### Study Design

This study was a small pilot project. The research team employed a qualitative descriptive approach to explore violently injured Black men’s perception on how they label themselves post firearm injury. This qualitative method allows firearm violence survivors to describe their experiences in their own words and give a rich description of their experiences and responses post injury [36].

### Sample

The sample for this study was recruited from a hospital-based violence intervention program (HVIP) at a level one trauma center in a single city in the Northeast. Fifteen (15) Black males who survived non-fatal firearm injuries and who were current and former participants in the local HVIP were approached by their case managers; ten met the inclusion criteria and consented to complete the interview. Study participants needed to meet the following inclusion criteria at the time of the study: (a) identify as a Black/African American male, (b) be over 18 years old, (c) survived a firearm injury, (d) patient in the local HVIP over three months (e) speak English, (f) have access to a telephone/cellphone, and (g) agree to be audio-recorded during the interview. The study was approved by the Institutional Review Board (Pro2022000552) and a certificate of confidentiality (CoC) was obtained from the National Institute of Health to protect participants recorded interviews and transcripts. Verbal informed consent was obtained due to federal physical distancing guidelines during COVID-19.

### Procedure

Semi-structured, open-ended interviews were conducted with participants during summer 2020. The research team created and utilized a semi-structured interview guide to assist us with the interviews. All interviews were conducted over the phone due to federal physical distancing guidelines and lasted 30–90 min. Because of the pandemic, recruitment became challenging, as restrictions on in-person interactions required that recruitment take place via telephone. Telephone recruitment was utilized because the research team did not want to assume study participants had access to platforms such as Zoom or Webex. Study personnel asked participants open-ended questions about their labeling perceptions, using follow up probes as needed [37]. The overall questions asked was “After being injured, do you identify as a victim or survivor? What made you choose survivor, victim, both or neither?”. The open-ended questions allowed for study participants to reflect on their feelings about the survivor versus victim label and how they may see themselves as one of the labels or neither. Although ten interviews were conducted, reviews of the interview transcripts revealed that nine participants yielded sufficient data to develop themes. Participants were provided a $50 incentive for their time [38].

Interviews were audiotaped, transcribed verbatim, and de-identified with multiple cross checks to ensure accuracy. Interviews were first, hand coded individually by three members of the research team, all three emerging research scholars due to the low number of interviews. After, the three coders collectively agreed on which codes to use. We used constant comparative analysis [39, 40] to ensure consistent interpretation of the statements and underlying themes of de-identified interviews. To maintain transferability, a careful record of procedure and rationale for coding was recorded in an audit trail [41]. The research team employed frequent debriefing and regular discussion to increase credibility [42] and inter-rater reliability [43]. Throughout all phases of data analysis and writing, the PI engaged in self-reflective documenting of study process and analytic decisions.

## Results

All the participants in this study were low-income, violently injured Black/African American males. Study participants disclosed their race and gender, self-identifying as non-Hispanic, African American/Black males. The men were between the ages of 21–44 years old and currently live or previously lived in a single urban Northeast city. Seventy percent (70%) of participants were injured in the same city as the hospital-based violence intervention program, while 30% were injured in adjacent urban cities. Of the participants, 20% reported being employed and having private insurance and 80% were unemployed with Medicaid. Twenty percent (20%) of participants reported being gang affiliated and were not asked to disclose their affiliation to a specific gang.

### Interview Findings

When asked “Do you consider yourself a survivor or victim?”, participants chose to accept or reject one or both labels we typically associate with surviving a firearm-related injury. Giving participants the opportunity to label themselves as they see fit gave the research team some insight on how they view societal labels. Majority of participants accepted the term “survivor” as these individuals associated “victim” with weak, while a few others embraced both labels while offering to us the understanding that they consider themselves partially a survivor because they physically survived. Lastly, two participants rejected both labels and decided to use terms they closely identified with as being part of the hood.

***“I never considered myself a victim…I am a survivor!”***

Several individuals self-subscribed to the label survivor. Descriptions of self as ‘strong-minded’ alluded to survival as an act of higher power. In addition, narratives of perseverance are consistent with both resilience and posttraumatic growth.

*“I never considered myself a victim. I could say, I’m a survivor because I survived a lot of stuff, a lot of different things I guess you would say are traumatic experiences, stuff that would cause people to probably have problems or stuff like that. I ain't going to say that just because I'm strong minded I don't let certain things get to me like that.”* (Dru, 28)

*“I’m a survivor cause I survived a lot of stuff. I done seen death in a lot of ways. I had a stroke- I almost died from that then the shooting incident and then the time when they found me in the graveyard not breathing. I picked survivor I guess because God got something planned for me and he ain’t want me to go yet.”* (Kevin, 40)

*“I consider myself a survivor because everything that's thrown at me, I managed to get through it, keep moving forward and just progressing my life.”* (Tommy, 36)

Another participant subscribed to the traditional definitions of survivor and victim as he associated survivor with agency and victim with passivity.

*“I’m a survivor. I survived them shots. I survived it. I'm never a victim because survivors are the people that are making it out of something and come out stronger. I think I gained a knowledge or a new appreciation* (Victor, 33)

For some, being a survivor is about physically, emotionally, and mentally surviving not only their injuries but anyadverse circumstances or events they had no choice but to confront. Participants narratives encourage us to consider survivorship in the context of lifetime adversities.

***“I feel like a victim…I’m still alive.”***

Two participants self-labeled themselves as both a victim and survivor but under different circumstances. The first participant saw himself as a both a physical victim and victim of a mainstream institutions.

*“I’m both. I feel like a victim because I feel like I ain't really got the help and then I feel like a survivor because I'm still alive. I feel like I could be a victim because when I did get shot there wasn't an investigation. I never spoke to the police. Nobody. Nobody even came, no cops ever came to the hospital or nothing. So, I feel like a survivor because I pulled through. I survived living in the streets with living in [urban neighborhood].”* (Stanley, 44)

His mentioning of the police points directly at him being a victim of structural violence. Due to the inability of the police acknowledging Stanley’s incident, he feels harmed by the structures put in place to ensure public safety.

*“I’m a little bit of both. I came out of it, okay. You know it could have been a lot worse. I was able to make that phone call to 911 and I'm here now. Somebody else, another innocent bystander was shot in that incident and didn't make it. He was pronounced on the scene. So yeah, I'm the survivor.”* (Tariq, 38)

Tariq and Stanley require us to balance both the vulnerability and the resilience of violently injured males. Interventions should consider the duality of violently injured males as a means of facilitating their recovery. An essential component of HVIPs, particularly those operating in marginalized communities, is to address structural violence in all its forms. Perception of oneself as a survivor is not made in a vacuum, but in the context of comparative outcomes of other victims of gun violence and within the background of structural violence.

“I consider myself, a f*cking warrior!”

Out of the ten participants, two participants rejected the survivor and victim labels as they saw themselves outside of those standard labels. One participant labeled himself as a warrior. He was physically and mentally engaged in the struggle of recovering after his firearm-related incident, and he persisted by getting better mentally as he stated he gained a new frame of mind and physically by learning how to walk again. Warrior symbolizes belonging to an honor bound belief system. Warriors in intergroup conflicts achieve nonmaterial rewards in the form of praise, reputational enhancement, social status, and admiration [44].

*“I consider myself a fucking warrior! I come from a long line of warriors! Write that down… A warrior! Well, you can say I got broken down and built back up. I got a new frame of mind. You can say that my survival was broke down. When I was in the hospital, it was rough. It was rough in the hospital because I had to learn how to walk again. You don't know if the situation [my health] is getting better. Doctors don’t want to tell you, you going to be alright. You know medically they can't tell you that. I've never been through nothing like that. I wouldn’t wish that on nobody but you gotta shake all those crazy feelings, you gotta shake that moping, worthlessness, and helplessness, it wasn’t your fault, guilt, you got to! Some people probably just weaker than others but it isn’t gone do you no good when you trying to rebuild.”* (Brayden, 35)

Another participant labeled himself as a casualty. A casualty can be understood as a person injured or killed, and it has a military connotation. He believes and accepts what happened to him comes with the territory of living in the hood.

*“I would consider myself neither. I just consider myself a casualty. It's the casualty of being in the hood. You know what I'm saying? See, I'm not seen as a civilian, I was a criminal. I acknowledge that and understand the fact that I was a criminal. When I was living a criminal lifestyle, I understood and accepted what came with that.”* (Reginald, 34)

Both ‘warrior’ and ‘casualty’ are traditionally used to describe participants of war, which also relates to how violence in concentrated places often are perceived as “battle zones” or “war zones” between individuals or groups [45], with its effects mirroring the hypervigilance, trauma, and mental/physical injuries associated with military personnel. In summation, given the small sample, these two participants with alternate labels suggest there are probably more variations identifications following firearm injury, which could be further explored among a different (e.g., geographic area, or ages, or recency injury) and/larger sample.

## Discussion

This study provides important insights into how violently injured Black men label themselves following a firearm-related injury. The findings reveal nuances in self-labeling that go beyond the binary of victim or survivor. Some participants embraced the survivor label due to overcoming their injuries and the adversity of urban street life. However, other participants acknowledged both survivor and victim labels, feeling they survived physically yet were victimized by lack of institutional support. Most noteworthy is that two participants rejected the conventional labels of victim and survivor, instead self-identifying as a “warrior” and “casualty.” This indicates that for some Black men, the typical framing of victim and survivor fails to fully capture their lived experience, labels, and identity following violent injury, and also alludes to military comparisons seen in the community firearm violence space.

A notable finding of this study is that none of the participants identified exclusively as victims following their violent injury. This diverges from existing research in adjacent fields showing victim self-identification does occur after experiences of interpersonal violence [2]. The complete absence of victim self-labeling in this sample may reflect influences of masculine socialization around strength and agency, as well as self-perceptions of manhood [46, 47]. Such internalized and socialized stigma can deter victim identification. Views of victimhood as carrying weakness and stigma may lead Black men to resist this label. Additionally, expectations of resilience against hardship due to racial stereotypes could discourage adopting the victim label. Further research should explore the social and psychological factors that lead violently injured Black men away from victim self-identification. The findings suggest complexity around notions of victimhood for this population that require deeper examination [48].

More research is needed to understand the factors that shape self-labeling for this population, including influences of masculine norms, racialized stereotypes, community context, and availability of services. This nuanced understanding could acknowledge both the interpersonal and structural nature of stressors faced by Black men that inform protective factors and methods of self-appraisal [49]. Racialized stereotypes of Black men as hypermasculine and superhumanly resilient may also influence injured Black men leanings toward survivor rather than victim identities [50]. Additionally, the community context of chronic violence and poverty in urban neighborhoods, along with distrust of historically oppressive systems and institutions (e.g., criminal legal system) [51], could potentially shape labeling.

The self-identification as a survivor versus victim following violent injury may have salient implications for the mental health of Black men. Internalizing a victim identity could exacerbate trauma reactions, negative cognitions about self-worth and powerlessness, feelings of helplessness, and other symptoms of depression and PTSD [52]. In contrast, adopting a survivor identity may facilitate resilience and post-traumatic growth by enhancing self-efficacy, hope, motivation to heal, and perceived control [53]. However, some scholars have cautioned that an exclusive focus on one term (e.g., survivorship) for a specific experience could pressure injured individuals to suppress vulnerable emotions or bypass trauma processing in efforts to appear strong and resilient [18, 47]. Furthermore, it is possible that premature adoption of a survivor identity may lead to denial or avoidance of the trauma's psychological impact, thereby impairing emotional healing. As seen through the sexual violence literature, for example, a larger dialogue around terms has increased in recent years, and there likely will not be consensus on a “best” term, as each person brings their own experiences and perceptions to their self-labels [28, 54]. As such, firearm violence policy and program efforts can suggest that the best way to identify someone who has experienced a firearm injury is to ask for, and then use, the term that each person prefers [55]. Efforts to reduce stigma are needed at multiple levels to prevent internalized victimhood among violently injured Black men. Specifically, public awareness campaigns could emphasize that surviving violence and seeking help require strength rather than weakness, while community partnerships could increase access to culturally congruent mental healthcare [56, 57]. Additionally, training healthcare providers in trauma-informed care and using sensitive language could mitigate re-traumatization [58]. Ultimately, broader policy reform is needed to address systemic marginalization across healthcare, criminal justice, education, and other institutions that fail Black men and communities. A multifaceted approach across these areas can shift social environments and schemas to reduce stigma and promote help-seeking behavior among violently injured Black men.

Given these complex considerations, providers, and social science researchers may be best served by rejecting a one-size-fits-all approach and allowing violently injured Black men to self-determine the self-appraisal that aligns most closely with their lived experience, needs, and cultural context [46]. Imposing labels could overlook individual differences and neglect the meaning-making process among those who have experienced a life-altering event. More research is needed to understand how social norms around masculinity, racial stereotypes, community context, and other sociocultural factors shape self-labeling following violent injury for Black men.

### Limitations

Given the small qualitative sample of Black men included in this study, there are limitations to extending the results of this study beyond the Black men who were not included as part of this data collection in connection with the HVIP, and among others who have experienced firearm injury [59]. Specifically, participants in this study enrolled in an HVIP and were participating in the HVIP or at least 3 months (or more). The experiences and life-views of these men may be different than those who were injured but did not enroll in the hospital’s HVIP and/or did not remain in the HVIP for at least 3 months. Transferability to other geographic locations, injury type, and/or demographic groups is also limited. Additionally, as noted in the methods section, data collection during COVID-19 limited collection to telephone contact and may have influenced the number of potential participants and participants willing to engage. Recruitment in person may have led to a higher sample size. Nonetheless, existing research into direct experiences of labeling and self-appraisal remains limited among this population that remains disproportionately afflicted by firearm violence [1, 18, 35].

## Conclusion

This study revealed self-labeling across the victim-survivor continuum and how the current dichotomy does not always capture Black men’s direct experiences. Future research examining the role of racially coded language, socio-demographic factors, and environmental contexts can further enlighten supportive and appropriate labels among people who have experienced firearm injury.
